# Mutations resulting in the formation of hyperactive complement convertases support cytocidal effect of anti-CD20 immunotherapeutics

**DOI:** 10.1007/s00262-019-02304-0

**Published:** 2019-02-06

**Authors:** Anna Felberg, Aleksandra Urban, Anna Borowska, Grzegorz Stasiłojć, Michał Taszner, Andrzej Hellmann, Anna Maria Blom, Marcin Okrój

**Affiliations:** 1 Department of Medical Biotechnology, Intercollegiate Faculty of Biotechnology, University of Gdańsk and Medical University of Gdańsk, Dębinki 1 Street, 80-211 Gdańsk, Poland; 2 Department of Hematology and Transplantology, Medical University of Gdańsk, Gdańsk, Poland; 3 Department of Translational Medicine, Lund University, Malmö, Sweden

**Keywords:** Complement, Immunotherapy, Rituximab, Ofatumumab, Chronic lymphocytic leukemia

## Abstract

Anti-CD20 monoclonal antibodies (mAbs) rituximab and ofatumumab are potent activators of the classical complement pathway, and have been approved for the treatment of B-cell malignancies. However, complement exhaustion and overexpression of complement inhibitors by cancer cells diminish their therapeutic potential. The strategies of targeting membrane complement inhibitors by function-blocking antibodies and the supplementation with fresh frozen plasma have been proposed to overcome tumour cell resistance. We present a novel approach, which utilizes gain-of-function variants of complement factor B (FB), a component of alternative C3/C5 convertases, which augment mAb-activated reactions through a positive feedback mechanism called an amplification loop. If complement concentration is limited, an addition of quadruple gain-of-function FB mutant p.D279G p.F286L p.K323E p.Y363A (or selected single mutants) results in significantly increased complement-mediated lysis of ofatumumab-resistant tumour cells, as well as the complete lysis of moderately sensitive cells. Importantly, this effect cannot be achieved by further increasing ofatumumab concentration. Potentiation of cytotoxic effect towards moderately sensitive cells was less apparent at physiological serum concentration. However, an addition of hyperactive FB could compensate the loss of cytotoxic potential of serum collected from the NHL and CLL patients after infusion of rituximab. Residual levels of rituximab in such sera, in combination with added FB, were able to efficiently lyse tumour cells. We suggest that the administration of gain-of-function variants of FB can restore cytotoxic potential of complement-exhausted serum and maximize the therapeutic effect of circulating anti-CD20 mAbs.

**Electronic supplementary material:**

The online version of this article (10.1007/s00262-019-02304-0) contains supplementary material, which is available to authorized users.

## Introduction

Rituximab, an anti-CD20 antibody, was the first antitumour mAb approved for clinical use. Although it is considered as a milestone in the treatment of B-cell lymphomas [1], there are reports of minimal response in patients, and thus many efforts aim to develop more potent anti-CD20 mAbs. Ofatumumab, the fully human anti-CD20 mAb, is already used in clinics, and a number of other candidates are passing clinical trials [2]. Ofatumumab, similar to rituximab, is classified as a type I mAb, which is a potent activator of the complement system [3]. Infusion of either rituximab [4] or ofatumumab [5] into CLL patients results in the rapid decrease of the classical complement pathway activity (CH50). However, post-infusion complement consumption does not automatically implicate tumour cell death, as numerous factors limit the efficacy of complement activation as an effector mechanism. The resistance of tumour cells to complement-dependent cytotoxicity (CDC) stems from overexpression [6, 7] and hijacking [8] of inhibitors normally protecting host cells from misguided complement attack. This makes the use of excessive concentrations of mAbs to overcome arrested CDC a suboptimal strategy. The first two components of the classical route, C1 and C2, are the ones with the lowest molar concentration in this pathway [9]. Given that most of the complement inhibitors target the mid-stages of the cascade, depletion of C1 and C2 by excessive mAbs may not only be unproductive in terms of decreased CDC but may also limit the effectiveness of further consecutive infusions [10]. Indirect evidence confirming complement exhaustion in vivo comes from observation in patients, in whom an addition of fresh frozen plasma to rituximab improved therapeutic effect [4, 11]. Moreover, high doses of anti-CD20 mAbs promote trogocytic removal of CD20 [12, 13] and produce population of CD20^low^ tumour cells. Therefore, maximizing the CDC potential of anti-CD20 mAbs by neutralizing complement inhibitors [7] and supplementation with exhaustible complement components [4] is a more rational strategy.

Ofatumumab (originally designed as 2F2) was first reported in 2004 as a fully human anti-CD20 antibody, which outperformed rituximab in CDC activation [14]. These results were later confirmed by Beurskens et al., who tested both antibodies within a wide range of tumour cell load and serum concentration [10] and also by our studies, in which in vitro susceptibility to CDC under limited availability of complement of eighteen CD20 + cell lines and fresh CLL cultures was assessed [15]. We found that ratio of the target (CD20) to complement inhibitor CD55 distinguished cells highly sensitive to both anti-CD20 mAbs from those of moderate sensitivity, which were killed more efficiently by ofatumumab. CD55 enhances dissociation of both classical and alternative complement convertases, nodal points in the complement cascade. Our results were in agreement with the work of Takei et al., who found that loss of CD20 and the concurrent increase of CD55 expression are attributes of acquired resistance to rituximab [16] and also in agreement with studies showing that CD20-positive cells of similar CD20 expression undergo CDC induced by rituximab to an extent dependent on their CD55 levels [17]. Neutralization of CD55 as a concept for increasing the efficacy of therapeutic mAbs was recently exploited by Macor et al., who tested bispecific antibodies targeting CD20 and CD55 in vitro and in mouse xenograft models of Burkitt lymphoma [18]. This concept was also further demonstrated when Mamidi et al. performed siRNA-mediated silencing of membrane complement inhibitors [19]. Notably, expression of complement inhibitors is not limited to tumour cells, but is ubiquitous within the human body. Therefore, there is a need for specific delivery of siRNA for this purpose. The affinity of bi-specific antibodies is restricted to only one type of membrane complement inhibitor, and does not cover the activity of other redundant inhibitors (e.g. factor H (FH)), which is present in serum in micromolar concentration and, when hijacked by tumour cells, contributes to anti-CD20 mAb resistance [8]. Being aware of these limitations, we propose a novel strategy, which combines supplementation with an exhaustible complement component while avoiding complement inhibitors. Instead of blocking inhibitors’ function, we utilized gain-of-function mutants of complement factor B (FB), which is a component of the alternative C3 and C5 convertases. Such mutants were identified in patients with atypical hemolytic uremic syndrome (aHUS) [20] and C3 glomerulopathies (C3G) [21] or designed in silico and shown experimentally [22] to form convertases insensitive to decay by multiple complement inhibitors.

## Materials and methods

### Protein expression and purification

Wild-type FB cDNA sequence (accession number NM_001710) additionally containing six histidine codons at 3′ terminus, as well as sequences for D279G, F286L, K323E, Y363A variants, and the quadruple mutant containing all aforementioned substitutions were codon optimized, synthesized and cloned into pCEP4 vector in the framework of GeneArt Gene Synthesis service by Thermo Fisher. Proteins were expressed and purified as described [23]. Briefly, vector DNA was transfected into HEK293 Freestyle cells using Freestyle Max reagent (Thermo Fisher). Conditioned FreeStyle 293 expression medium (Thermo Fisher) was collected at days 2, 4 and 7 post-transfection and stored at − 80 °C until the protein purification. The resulting proteins were purified with HisTrap FF affinity column (GE Healthcare) and elution was carried out with an imidazole gradient.

### In vitro culture of CD20-positive cells

All cell lines were cultured in RPMI 1640 medium with l-glutamine (Mediatech) supplemented with 10% foetal bovine serum (PANBiotech) at 37 °C and humidified 5% CO_2_ atmosphere.

### Clinical material (serum and erythrocytes)

Serum samples were collected from five patients admitted to Dept. of Hematology, Medical University of Gdańsk. The inclusion criterion was a diagnosis of B-cell malignancy with no prior treatment with anti-CD20 mAbs. Patients #1 and #3 were diagnosed with diffuse large B-cell lymphoma (DLBCL), patients #2 and #4 with follicular lymphoma and patient #5 with CLL. Blood was collected into Vacutainer tubes with clot activator (BD Biosciences) before and after the first intra-venal infusion of standard rituximab dose (375 mg per 1 m^2^ of body surface). Isolated blood was left in room temperature until clot formation (around 20 min), then centrifuged at 700 × *g* for 12 min at 4 °C, pooled, centrifuged again to get rid of residual cells, aliquoted, and finally stored at − 80 °C until needed. The same procedure was applied for blood collection from healthy volunteers used for the preparation of normal human serum (NHS) as described elsewhere [24]. For human erythrocytes, blood was collected into K_2_EDTA Vacutainer tube (BD Biosciences), then loaded onto a gradient of Histopaque-1077 (Sigma) and centrifuged. The erythrocyte-containing fraction was collected, washed 5 × with PBS buffer, suspended 1:1 in Alsever’s solution, and kept at 4 °C until the experiment.

### Functional assays

Hemolytic assay assessing the ability of factor B mutants to enhance classical complement pathway was performed as described [25]. In some of the assays, factor B-depleted serum (Δ FB, Complement Technologies) was used instead of NHS. Two-step convertase assays measuring convertase activity over the time were performed as in [25]. Briefly, rabbit erythrocytes (Centre of Experimental Medicine, Silesian Medical University, Poland) were subjected to 5% normal human serum supplemented with wild-type or mutated factor B and C5 blocker (OmCI) for the indicated period of time. Cells were then washed and guinea pig serum (Harlan Laboratories) diluted 1:40 v:v in 40 mM EDTA-GVB (gelatin veronal buffer) buffer was added to develop lytic sites from convertases preformed in the first step of the experiment. Hemolysis was proportional to convertases’ activity at given time point. A hemolytic assay measuring bystander lysis of human erythrocytes was performed by co-incubation of 1 × 10^5^ ofatumumab-sensitized Raji cells in 10% or 50% NHS, optionally supplemented with 20 µg/ml of wild-type or mutated FB. The amount of erythrocytes was adjusted in a way that full lysis sample (10 µl of erythrocyte solution + 90 µl of water) gave absorbance readout of 2.0 AU at 405 nm. Quantification of released haemoglobin was assessed after 30 min.

### CDC assay

CD20-positive cells were harvested, suspended in complete medium to yield 10^6^ cells/ml and calcein-AM (Sigma) was added to the final concentration of 1 µg/ml. After 30 min incubation at standard culture conditions, cells were washed with PBS buffer with Ca^2+^/Mg^2+^ (Biowest), loaded into the V-shape wells of 96-well microplate (Nunc) at 10^5^ cells (or more, as indicated separately in the text) per well and pelleted. Pellets were overlaid with PBS w. Ca^2+^/Mg^2+^ containing desired concentration of ofatumumab (GlaxoSmithKline) and NHS, in a total volume of 50 µl. Microplates were incubated for 30 min. at 37 °C and shaken at 650 rpm, then overlaid with another 50 µl of PBS buffer and centrifuged. Eighty microliter of the supernatant was transferred into flat-bottom plate and fluorescence 485/515 nm was measured in Synergy H1 (Biotek) reader. Fluorescence readout obtained for cells loaded with calcein-AM and lysed with 2% NP40 (Sigma) was considered as full lysis.

### Assays measuring complement consumption/complement activity restoration

The concept of complement consumption assay was similar to that originally described by Beurskens et al. [10]. One hundred thousand cells of the selected CD20-positive cell lines (Daudi and Raji) were harvested and suspended in PBS solution with Ca^2+^/Mg^2+^-containing NHS (5% for Daudi, 10% for Raji cells) and ofatumumab (50 µg/ml). Some solutions were additionally supplemented with their physiological concentration of recombinant wild-type or quadruple FB mutant. Cells were incubated at 37 °C and 50 µl of the sample was pelleted after selected time points (0.5, 1, 2, 4, 24 h). Supernatants were collected and used in CDC assay (performed as described above) instead of an aliquot of fresh NHS. In alternative versions of the assay aimed to assess the restoration of cytotoxic potential by mutated FB, the first step was carried out in the presence of 5 × 10^5^ Raji cells and 50% of NHS but without addition of FB. Then supernatant was transferred to the new portion of calcein-AM-labelled Raji cells (1 × 10^5^) and FB variants were added.

## Results

### Alternative pathway plays a role in anti-CD20 mAb-mediated complement activation

**Fig. 1:**
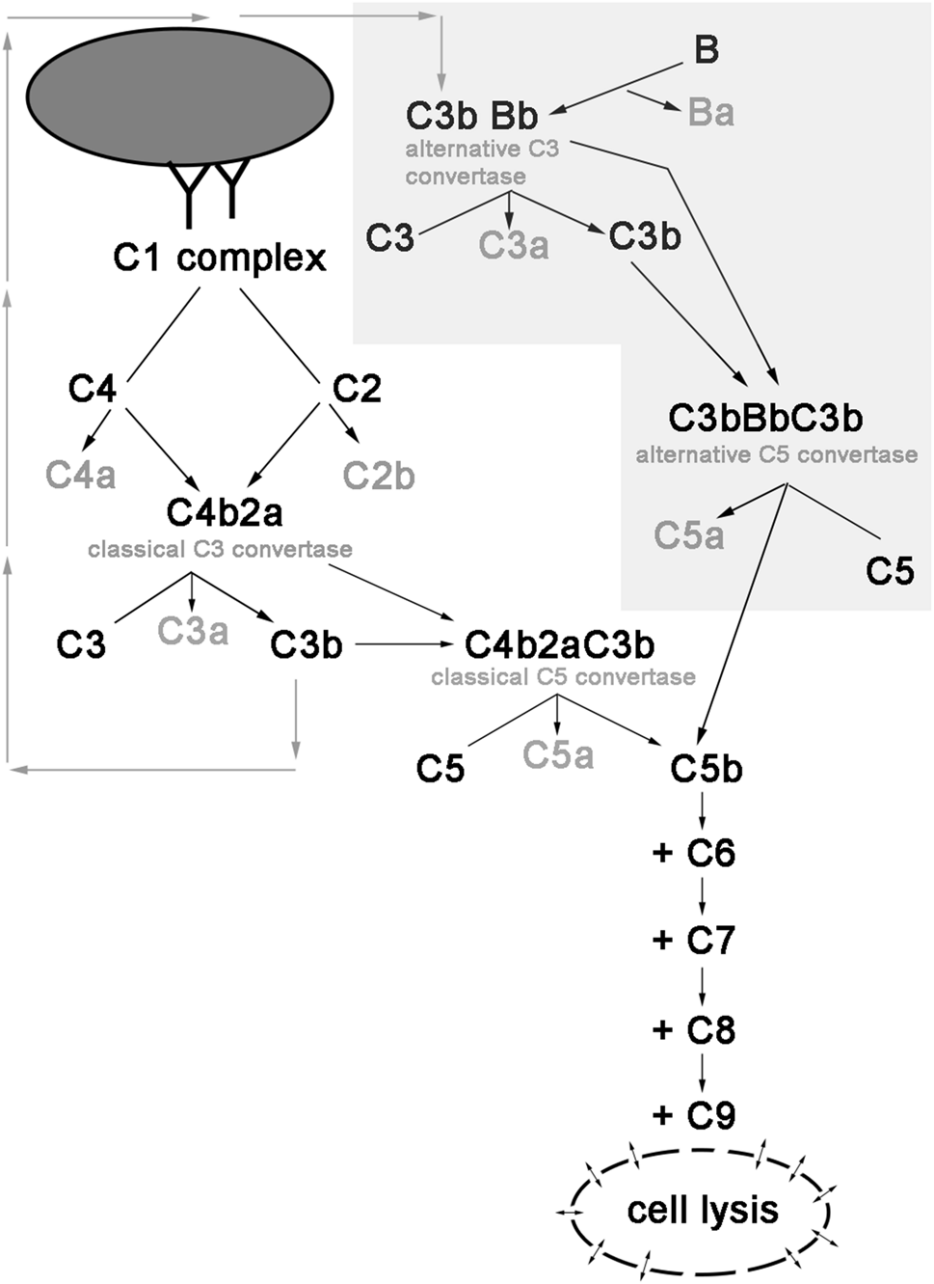
Scheme of mAb-initiated complement activation. Binding of antibodies onto the surface of target cell initiates the classical complement pathway. Classical C3 convertase cleaves C3 molecule into C3a and C3b fragments. Binding of C3b to the parental C3 convertase complex switches its substrate specificity to C5, hence forming C5 classical convertase. Alternatively, binding of C3b directly to the cell surface launches so-called amplification loop (shaded polygon), whereby interaction with factor B leads to the formation of alternative C3 and C5 convertases. Cleavage of C5 by either classical or alternative C5 convertase initiates common, terminal pathway eventually leading to osmotic lysis of target cell

**Fig. 2:**
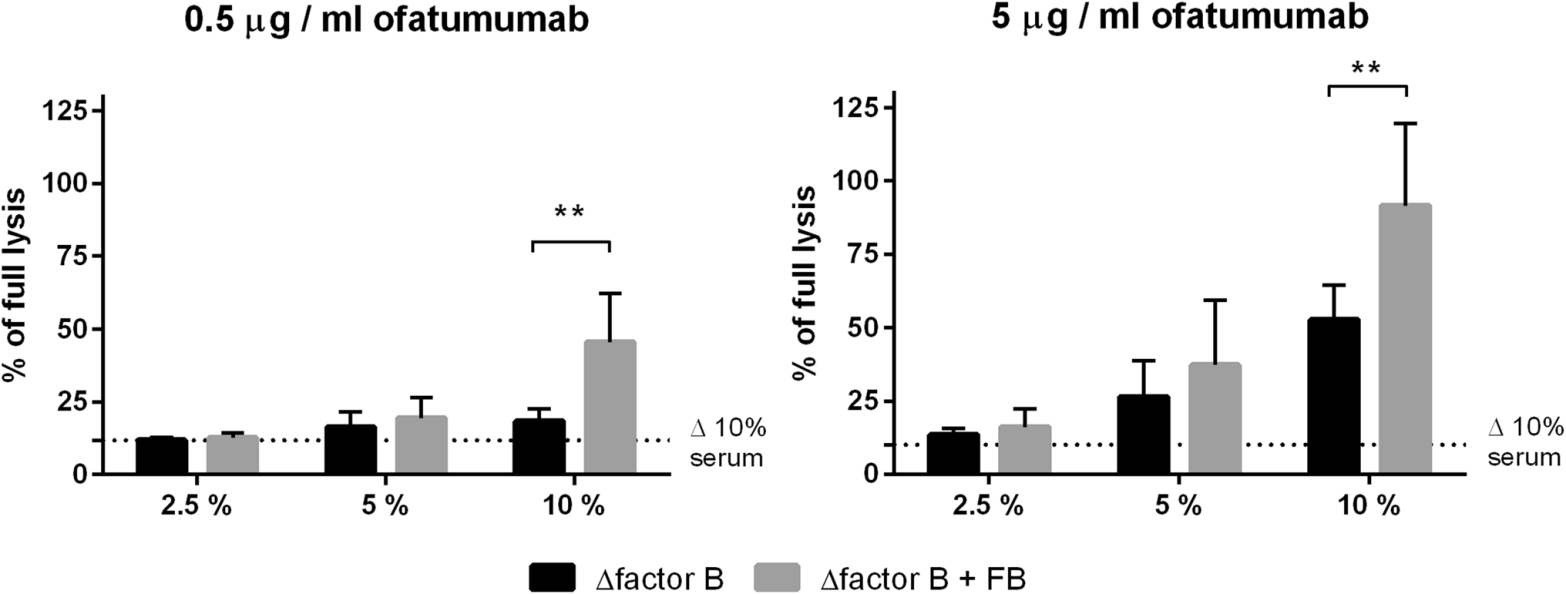
CDC exerted by ofatumumab in serum ± FB. Calcein-AM-labelled Daudi cells were treated with ofatumumab (0.5 µg/ml or 5 µg/ml, left and right panel, respectively) and 2.5%, 5% and 10% of FB-depleted serum or the same serum reconstituted with recombinant wild-type FB. The readout of cells lysed with 2% NP40 was considered as maximal (100%) lysis whereas readout obtained in 10% of heat-inactivated normal human serum (Δ 10%) was considered as a background, complement independent lysis and depicted by dotted line. The graphs present data from three independent experiments and error bars represent standard deviation. **Statistical significance between FB-deficient and sufficient serum at *p* level < 0.01 according to Sidak’s multiple comparison test for paired data (GraphPad Prism)

While antitumour antibodies initiate classical complement pathway, augmentation of the cascade is achieved by the amplification loop, which engages alternative convertases and FB (Fig. 1). To confirm that alternative complement pathway is relevant to the amplification of anti-CD20 mAb-mediated complement activation, we performed CDC assay in serum with or without FB. Previously, we found Daudi cells to be highly sensitive to ofatumumab at low serum concentrations [15] and now we tested the same cells at two different, suboptimal concentrations of ofatumumab to better visualize the importance of alternative pathway. In parallel with increasing serum concentration, there was a trend towards higher CDC in FB-reconstituted serum. Differences reached the twofold level and statistical significance (*p* < 0.01) at 10% serum (Fig. 2) confirming that amplification of the classical complement pathway via formation of alternative convertases plays an important role even in case of cells highly sensitive to ofatumumab.

### Gain-of-function mutants of factor B enhance cytotoxic effect of anti-CD20 mAbs by supporting activity of complement convertases

We successfully expressed and purified wild-type and D279G, K323E, Y363A single mutants but we could not express the F286L mutant, in spite of three independent attempts. Notably, we obtained quadruple mutant, which embraced D279G, K323E, Y363A and F286L substitutions (supplementary Fig. 1). Aforementioned mutations in factor B were previously characterized as providing resistance to numerous complement inhibitors (FH, CR1 and CD55), enhanced C3 turnover and binding affinity of FB to C3b fragment [20, 22, 26]. Next, we tested whether these FB mutants have potential to enhance CDC. To do so, we performed a preliminary study on antibody-sensitized sheep erythrocytes, a common model for assessment of the classical complement pathway [25]. The experiment was performed in FB-depleted serum reconstituted with increasing concentrations of given FB variant. Addition of all recombinant FB mutants but Y363A caused significantly more intense hemolysis, comparing to addition of wild-type (WT) FB (supplementary Fig. 2).

**Fig. 3:**
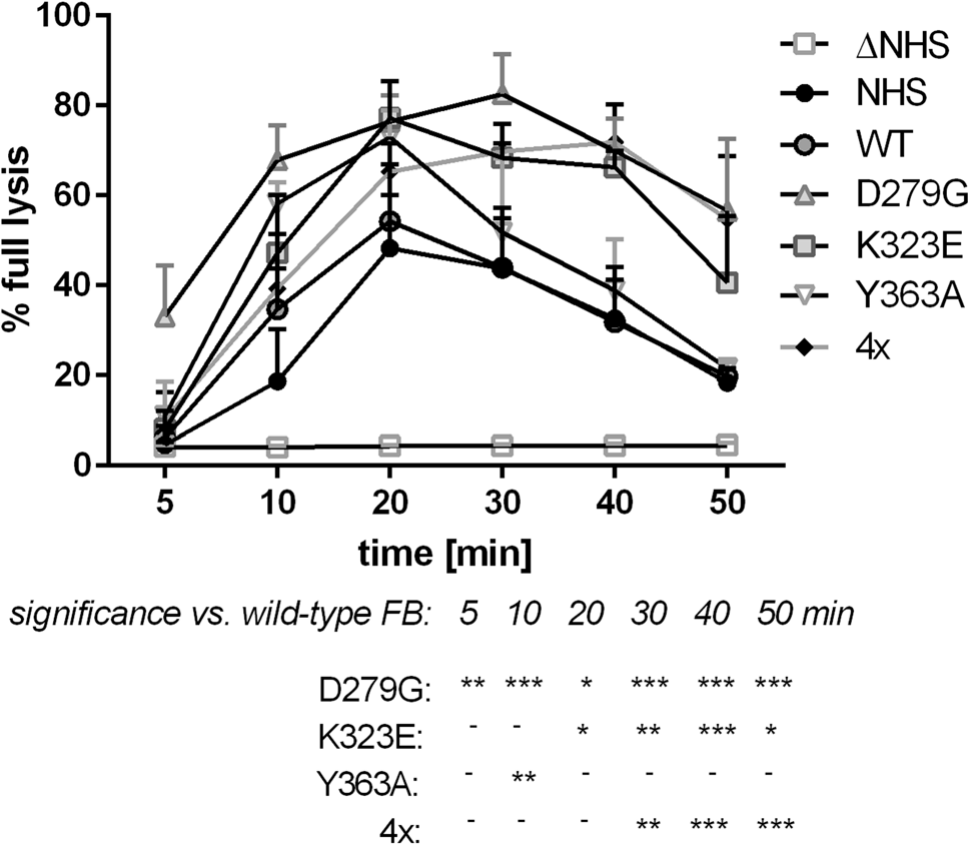
Activity of alternative complement convertases formed in serum supplemented with mutated FB variants. Assay was performed with 5% NHS supplemented with 10 µg/ml of wild-type (WT) or single (D279G, K323E or Y363A) or quadruple (4×) gain-of-function FB mutant. Heat-inactivated normal human serum (Δ NHS) was used as a negative control. Data are collected from three independent experiments and error bars show standard deviation. Statistical significance was assessed at *p* < 0.05*, *p* < 0.01** or *p* < 0.001*** according to Dunnett’s multiple comparison test (GraphPad Prism)

The hemolytic assay shows the overall effect of complement activation but provides no information about the efficiency of particular steps of the cascade. Therefore, we ran an experiment to find out whether an addition of FB mutants to NHS results in formation of alternative complement convertases of elevated activity or extended stability. This experiment was performed on the surface of rabbit erythrocytes, which spontaneously activate alternative complement pathway [25]. Serum supplemented with FB variants D279G and K323E formed alternative convertases of either higher activity (as visualized by more intense hemolysis at *T*_max_ point = 20 min) or extended stability (as the decay of convertase activity is slower than upon addition of wild-type FB) (Fig. 3). Convertases formed in the presence of D279G and Y363A mutants reached their high activity significantly faster; however, in case of Y363A mutation such effect was visible only at single time point of 10 min. The addition of quadruple mutant resulted in the formation of convertases of extended stability, as decay rate was significantly slower.

**Fig. 4:**
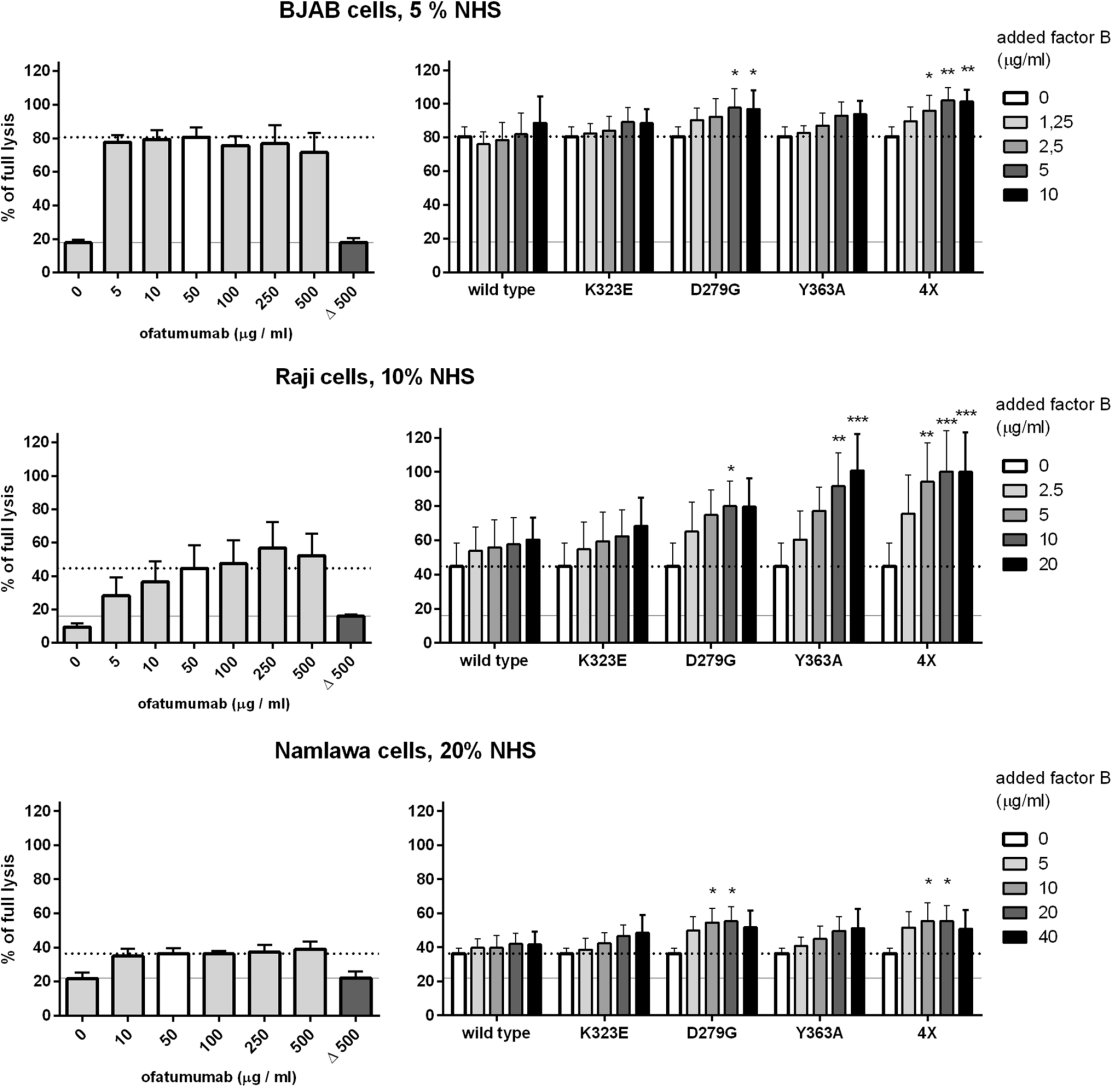
CDC of ofatumumab supplemented with gain-of-function variants of FB in CD20 + cells. The left panel shows CDC exerted by ofatumumab in BJAB, Raji and Namalwa cells. The last, dark grey bar (Δ 500) and grey solid line show the readout obtained in heat-inactivated serum, thus considered as a background, complement-independent lysis. The bar for 50 µg/ml is indicated with white colour since this concentration was applied in experiments shown in right panel (and this reference CDC level is further indicated with dotted line). Cells were treated with ofatumumab, serum (at the concentration indicated for each cell line) and supplemented with increasing concentrations of wild-type or gain-of-function single or quadruple FB mutant. Statistical significance at *p* level < 0.05*, *p <* 0.01** and *p* < 0.001*** in comparison to CDC without any additional FB (white bar) is calculated according to Dunnett’s multiple comparison test (GraphPad Prism). Graphs present data from three or four (BJAB cells) independent experiments and error bars show standard deviation

Erythrocytes are a widely used model for complement system activation analysis, but it may not reflect all physiologically important issues since the lack of human complement inhibitors may lead to underestimation of some relevant effects. To get full insight into consequences of each mutation on mAb-mediated killing of tumour cells, CDC assay employing anti-CD20 mAb (ofatumumab) was performed. Based on our previous work, we selected three cell lines of high (BJAB), moderate (Raji) and low (Namalwa) sensitivity to ofatumumab-mediated CDC [15]. Eighty percent of BJAB cells lysed already at 5% serum and 50 µg/ml ofatumumab. Addition of D279G mutant significantly increased CDC (*p* < 0.05) and addition of quadruple mutant increased lysis to maximal level (*p* < 0.01). Importantly, this effect could not be achieved by further increase of mAb concentration (Fig. 4, upper panel). CDC of Raji cells at 10% serum and 50 µg/ml ofatumumab oscillated around 45% of full lysis and further addition of ofatumumab slightly but not statistically significantly increased CDC (Fig. 4, middle panel). However, supplementation with D279G, Y363A and quadruple FB mutants resulted in significant increase of CDC and the two latter proteins brought CDC to maximal level. Notably, as little as 5 µg/ml of quadruple mutant (corresponding to c.a. 25% of wild-type FB content in 10% serum) caused more than double increase of CDC. The supportive effect of D279G and quadruple mutant was also observed in case of Namalwa cells normally resistant to ofatumumab and increased CDC from 36 to 55% of full lysis in 20% serum (*p* < 0.05, Fig. 4, bottom panel).

**Fig. 5:**
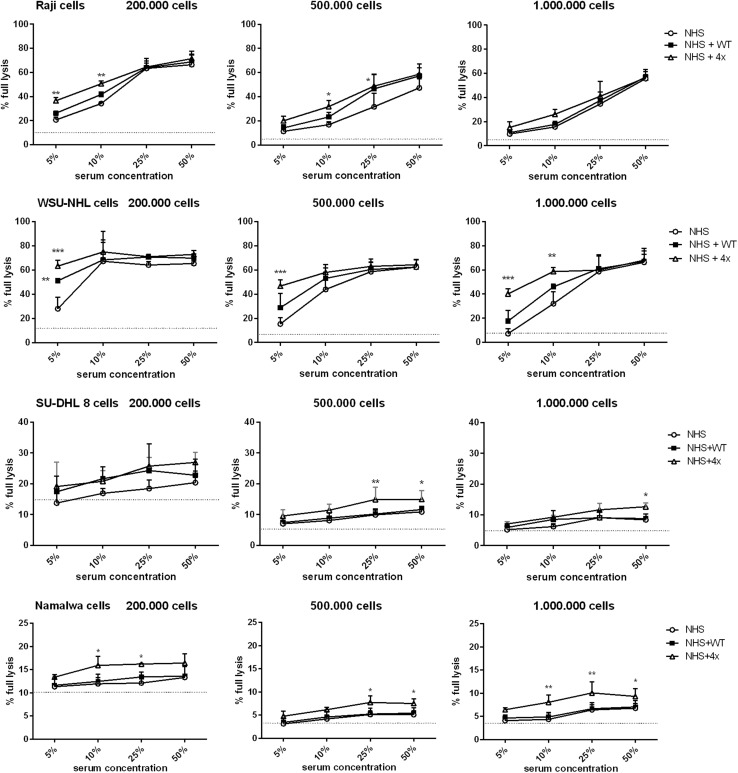
CDC mediated by ofatumumab supplemented with quadruple FB mutant in CD20 + cells—titration of serum and cell number. The graph shows CDC mediated by ofatumumab in WSU-NHL, Raji, Namalwa and SU-DHL-8 cells upon different serum concentrations and number of cells per well. The quadruple gain-of-function mutant or the wild-type FB was added to respective samples at a concentration of 20 µg/ml. Grey solid line shows the readout obtained in 50% heat-inactivated serum, thus considered as a background, complement-independent lysis. Statistical significance at *p* level < 0.05*, *p* < 0.01** and in comparison to CDC without any additional FB is calculated according to Dunnett’s multiple comparison test (GraphPad Prism). Graphs present data from three independent experiments and error bars show standard deviation

Experiments presented in Fig. 4 were performed at serum concentration lower than physiological (50%), thus modelling conditions of limited complement availability. We checked whether the same CDC-enhancing effect of quadruple mutant could be observed at physiological serum concentration. Further, CDC assays were performed using a higher number of tumour cells (2 × 10^5^, 5 × 10^5^and 1 × 10^6^ per well) in a range of serum concentration between 5% and 50% (Fig. 5). Next to previously analysed Raji and Namalwa cells, we also incorporated two other cell lines: moderately sensitive (similar to Raji) WSU-NHL cells and resistant (similar to Namalwa) SU-DHL-8 cells. As expected, overall CDC decreased with the increase of cell number. Importantly, CDC-enhancing effect of quadruple mutant on moderately sensitive cells was only detected at lower (5% and 10%) serum concentrations while diminished at physiological serum concentration (Fig. 5, upper two panels). In contrast, the effect on resistant cell lines was statistically significant upon physiological serum concentration but probably of limited biological relevance, as the overall percentage of lysis did not exceed 25% and 15% of tumour cells, respectively (Fig. 5, bottom panels).

### Gain-of-function FB mutants can restore cytotoxic potential of serum after complement consumption

**Fig. 6:**
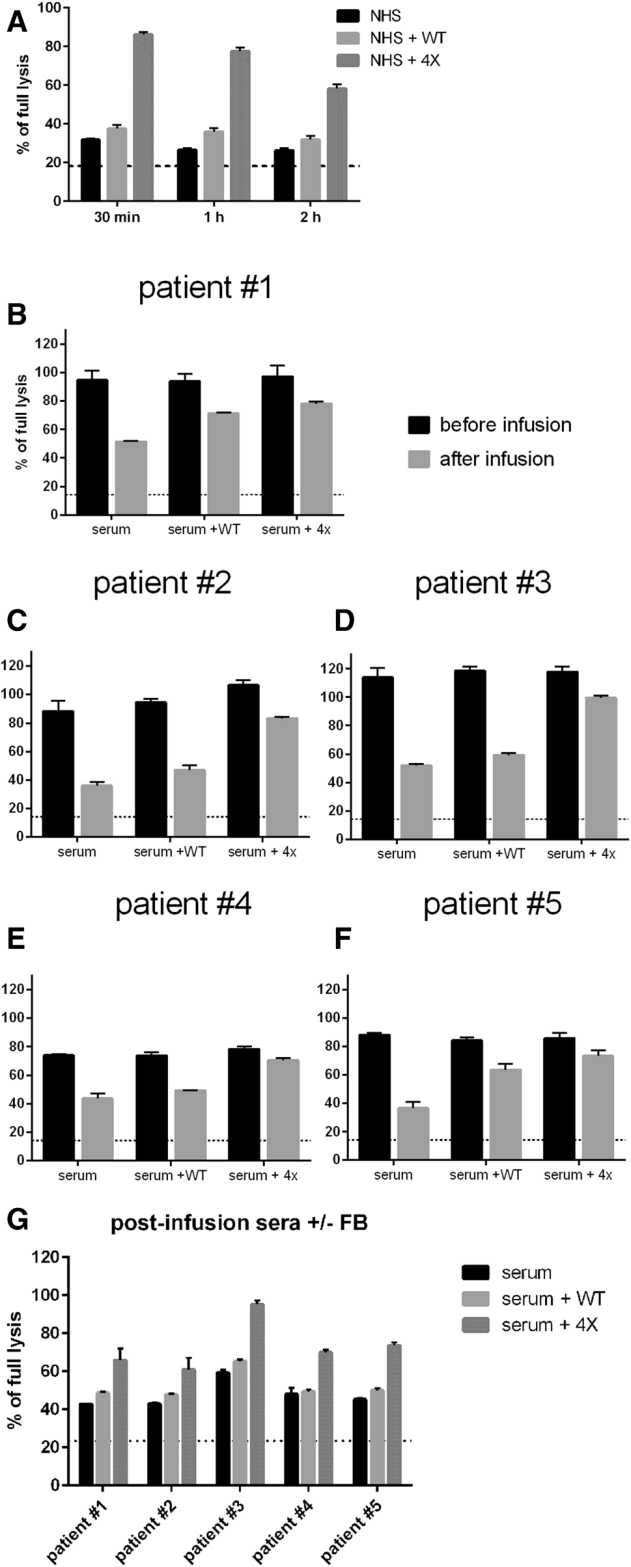
Effect of quadruple FB mutant on CDC potential of complement-depleted sera. Panel **a** 50% NHS was incubated with ofatumumab-sensitized Raji cells (5 × 10^5^) for 30, 60 or 120 min and thereafter supernatant was transferred to the new portion on calcein-AM-labelled Raji cells (1 × 10^5^). 20 µg/ml of FB variants was added at this point and CDC was measured. The graph shows one representative (out of two performed) experiment, each analysed in triplicates. Panels **b**–**f** show CDC readouts of patients’ sera collected before and after the first infusion of rituximab. Sera were supplemented with 50 µg/ml of ofatumumab and optionally with 20 µg/ml of FB variants. Panel **g** shows the CDC of post-infusion sera with no addition of ofatumumab and optionally supplemented with 20 µg/ml of FB variants. Experiments shown in panels b–f were run in triplicates

It is known that administration of anti-CD20 mAbs induces rapid depletion of complement and possibly leaves the body devoid of effector mechanism capable of further killing of tumour cells [4–6, 10]. Therefore, we decided to model hypothetical conditions of exhausted complement in patient’s serum. Two-step CDC assay was performed [10]. In the first step, complement in 50% serum was subjected to consumption by ofatumumab-sensitized cells and then in the second step, the supernatant was transferred to the fresh portion of tumour cells labelled with calcein-AM. Results showed that an addition of quadruple FB mutant at the second step but not wild-type FB can rescue cytotoxic activity of serum (Fig. 6a). The same was proven for patients’ sera collected before and after infusion of rituximab. Addition of quadruple FB mutant to 50% pre-infusion serum supplemented with ofatumumab had little effect on CDC but substantially improved CDC potential of post-infusion sera, which otherwise lost half of their activity (Fig. 6b–f). Notably, the same result was obtained for sera from all five patients with different diagnoses of B-cell malignancy. Finally, we showed that quadruple mutant can even boost the CDC potential of post-infusion sera without addition of extra ofatumumab, i.e. making use of rituximab remaining in these sera (Fig. 6g) thus showing that not the availability of antitumour mAb but availability of complement is a factor limiting effective CDC of tumour cells in patients’ sera.

### Complement consumption and bystander lysis upon the addition of gain-of-function variants of FB

**Fig. 7:**
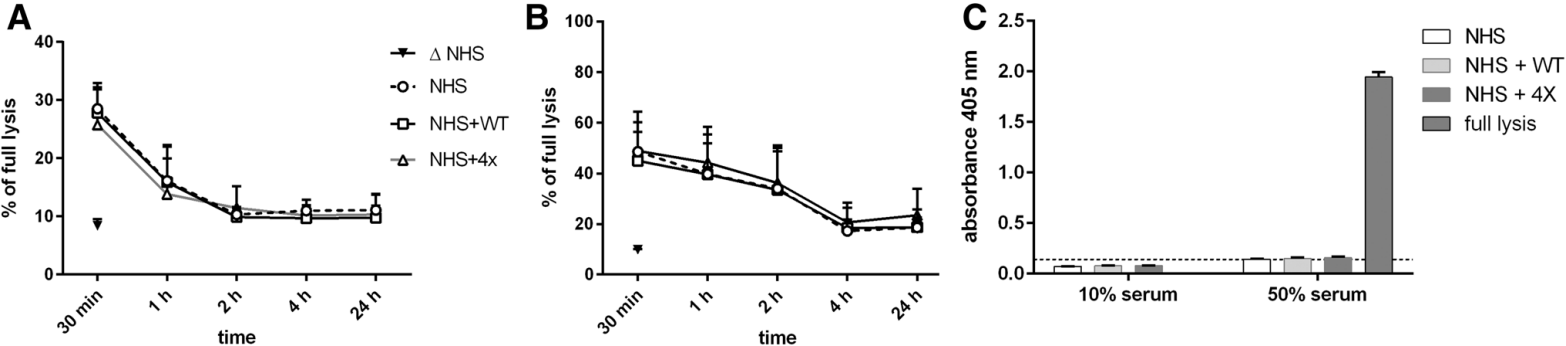
Evaluation of unwanted side effects upon serum supplementation with wild-type FB and quadruple gain-of-function mutant. Raji **a** and Daudi **b** cells were treated with ofatumumab and NHS for the indicated time period. Some serum samples were supplemented with wild-type FB (WT) or quadruple FB mutant (4×). Afterwards, supernatants were collected and used instead of fresh serum sample in regular CDC assay performed on calcein-AM-loaded cells. The readout obtained for sample of heat-inactivated serum incubated for 30 min is shown as negative control. Panel **c** Raji cells were co-incubated with human erythrocytes, NHS, ofatumumab and optionally, wild-type or quadruple FB mutant. Absorbance of sample where water was added instead of serum solution was considered as positive control (full lysis). Data are collected from three independent experiments and error bars show standard deviation

We showed that gain-of-function components of alternative convertases can enhance cytotoxic effect of antitumour antibodies. However, due to the nature of alternative pathway, which is constantly active at low level, these proteins boost spontaneous C3 activation and may deplete the available pool of exhaustible complement components. Accordingly, hypocomplementemia is often the hallmark of mutations leading to overactive phenotype of alternative convertases [27, 28]. Therefore, we performed functional assay comparing the ratio of CDC decay in serum without any supplements and sera supplemented with wild-type FB or quadruple gain-of-function FB mutant (which best supported CDC in cytotoxic assays). Concentrations of added FB corresponded to maximal concentrations used in CDC assay shown in Fig. 4. Raji or Daudi cells were incubated with ofatumumab and serum ± supplements for time periods varying from 30 min to 24 h. Putative complement consumption influences cytotoxic capacity of serum, which was evaluated in the next step of the experiment. Supernatants were collected and used instead of serum in another round of CDC assay performed on calcein-AM-loaded cells. CDC dropped gradually from time point of 30 min and reached the lowest level after 2 h (Raji) or 4 h (Daudi). Importantly, in our experimental conditions, we did not notice any significant differences in CDC decay between normal human serum and sera with supplements (Fig. 7a, b). Thus, it is theoretically possible to adjust concentration of FB mutants in a way, which would enable enhancement of type I mAb-mediated cytotoxic effect but eliminate excessive depletion of complement in the longer period of time. However, we have evidence that such problem may exist when the most active variants of FB dominate in the sample. Our pilot hemolytic and convertase assays were performed in FB-depleted serum reconstituted with recombinant FB mutants. In such conditions, hemolytic activity of quadruple mutant was negligible (supplementary Fig. 3) and convertase activity (supplementary Fig. 4) was diminished in terms of amplitude and delayed in terms of *T*_max_ point. Also, we have tittered the concentration of quadruple FB mutant at constant serum concentration and observed that the increase of FB beyond 20 µg/ml did not cause more efficient CDC whereas 100 µg/ml concentration decreased CDC (data not shown). Together with the decrease of CDC-enhancing potential of quadruple mutant when moderately sensitive cells were incubated at high serum concentration (Fig. 5), these data suggest unproductive complement consumption in the fluid phase when either too many molecules of hyperactive FB are present or too many lytic sites are available.

Another hypothetic unwanted side effect of complement activation is bystander lysis, a phenomenon caused by soluble, initial components of the terminal complement pathway, i.e. C5b6 complex. This intermediate can be inserted into the membrane of virtually all cells, not necessarily the cell, which initiated complement activation and enables the formation of Membrane Attack Complex (MAC). Extensive complement activation is a condition favourable for such unspecific cell damage [29]. We tested whether bystander lysis can be provoked by the co-incubation of ofatumumab-sensitized Raji cells and human erythrocytes with either 10% or 50% serum in the presence of wild-type or mutated FB (Fig. 7c). We observed very little hemolysis comparable to the level obtained for sample incubated with heat-inactivated serum (negative control). There were no differences between wild-type, mutated FB (added at the concentration optimal for enhancement of complement-mediated lysis) and serum sample without additions (Fig. 7c).

## Discussion

Our results provide a proof of concept that hyperactive variants of convertase forming proteins capable of bypassing multiple complement inhibitors can act as supporters of type I anti-CD20 mAbs. Notably, CDC assays performed on CD20 + cells revealed that results of hemolytic assays and convertase assays performed on erythrocytes do not always correspond to the results of experiments performed on human tumour cells. For example, Y363A mutation, described as the one resulting in the formation of convertases resistant to the inhibitory effect of CD55 [22] caused no increase in hemolysis of sheep erythrocytes (supplementary Fig. 2) and only moderate effect on alternative convertases (Fig. 3). Conversely, when used as a supplement to ofatumumab in CDC assay, it showed extraordinary enhancement of complement-mediated killing of Raji cells (Fig. 4) upon limited availability of complement. One possible explanation is that sheep erythrocytes are not equipped with human complement inhibitors but their homologues. Another explanation involves cell line-specific expression of target molecule (CD20) and complement inhibitors (e.g. CD46, CD55, CD59). Previously, we showed that BJAB, Raji and Namalwa cells used in our model experiments are characterized by different ratios of these surface proteins, which influence their sensitivity to anti-CD20 mAbs [15]. This is why we tested quadruple mutant embracing several gain-of-function mutations. Such protein should be more universally useful towards different types of tumour cells. Indeed, quadruple mutant performed better than individual single mutants in most of the CDC assays and its effect was visible at lower concentrations. However, this conclusion is limited by inability to produce single F286L mutant in our expression system.

We revealed that the quadruple gain-of-function mutant may exert different effects on CDC, depending on target cell sensitivity to ofatumumab and serum concentration. It can significantly (but still at low overall level) enhance CDC of ofatumumab-resistant cells under physiological serum concentration but the same effect on moderately sensitive cells is detectable only under low serum concentration. Nonetheless, we postulate that such a model is still relevant to the field and adequate for a situation where complement is quickly exhausted following the administration of antitumour antibodies to the bloodstream of patients with a high tumour burden. As evidenced by our experiments using clinical samples collected from DLBCL, CLL and follicular lymphoma patients treated with rituximab, an addition of quadruple gain-of-function FB mutant rescues cytotoxic capacity of their post-infusion sera (Fig. 6b–f) and helps to efficiently utilize remaining rituximab for killing tumour cells (Fig. 6g). Possibly, supplementation with gain-of-function FB mutants can be considered as a way to combat tumour cells, which escaped the first wave of CDC following administration of anti-CD20 mAbs. Also, in the same context it is worth mentioning that around 40% of patients with CLL have diminished baseline complement activity [30]. Another group reported that ex vivo experiments with CLL cells show significantly lower anti-CD20 mAb-mediated lysis in the presence of autologous patients’ serum than that with normal human serum [31].

Under physiological conditions, complement convertases decay rapidly in intrinsic and extrinsic processes. On the one hand, higher and longer activity of convertases creates more lytic sites on the surface of target cells. On the other hand, increase of convertase half-life raises a chance for misguided deposition of active complement components on self-cells and tissues. Indeed, some carriers of gain-of-function mutations in alternative convertase constituents develop autoimmune diseases [20, 21] and, therefore, use of hyperactive FB may bring safety concerns. However, these FB mutations show incomplete penetrance [20] and, in contrast to autoimmune patients constantly exposed to etiologic factors, suggested supplementation with gain-of-function mutants would be temporary and coordinated with anti-CD20 mAb infusions. Administration of type I anti-CD20 antibodies into, e.g. CLL patients, who typically carry from 3 × 10^4^ to 3 × 10^5^ malignant B cells per microliter [32], causes massive complement engagement. With such a high tumour burden, hyperactive convertase components should incorporate into enzymatic complexes formed on target cells rather than those formed due to incidental, misguided complement activation. We showed that in vitro supplementation of NHS with quadruple FB mutant (up to the concentration of 20 µg/ml, which was effective in enhancement of CDC) does not provoke excessive and unproductive complement consumption, which normally is one of the unwanted side effects of gain-of-function components of alternative convertases. We also did not observe any bystander lysis of human erythrocytes co-incubated with ofatumumab-sensitized Raji cells and quadruple FB mutant added at the optimal concentration for enhancement of CDC. Our choice of erythrocytes as a model was due to erythrocytes being the most abundant component of blood compartments, and their relatively high vulnerability to osmotic lysis in comparison to nucleated cells. Moreover, intravascular hemolysis is a crucial event of diseases such as paroxysmal nocturnal hemoglobinuria (PNH) [33] or atypical hemolytic uremic syndrome (aHUS) [34], which are caused by malfunctions in the regulation of complement convertases. It is important to note that the risk of the development of complement-related diseases should be addressed in in vivo experiments in the animal model of B-cell malignancy, which we did not perform in this study. Another potential problem with proposed strategy is that patients receiving hyperactive FB variants may develop an immune response to these proteins and thus limit their efficacy. A rare character of such mutations and diseases related to them can explain the lack of reports on antibody response to altered FB molecules; however, the phenomenon is known for certain tumour-specific antigens, e.g. p53 mutants [35]. Nevertheless, by analogy to anti-p53 seropositive patients, efficient presentation of putative antigenic fragments of FB mutants to B lymphocytes would demand appropriate HLA class II alleles, thus limiting the chances for such unfavourable scenario to carriers of certain HLA haplotypes [35]. Although we acknowledge that unspecified risk related to an application of gain-of-function FB variants may exist, our aim was to provide a proof of concept for a novel strategy maximizing the efficacy of complement-mediated immunotherapeutics. Our experiments showed that application of gain-of-function FB mutants can compensate complement exhaustion in patients treated with rituximab. Moreover, addition of such proteins to post-infusion serum exploits the cytotoxic potential of the drug still present in patient’s bloodstream.

## Electronic supplementary material

Below is the link to the electronic supplementary material.

Supplementary material 1 (PDF 510 KB)

## Abbreviations

aHUS: Atypical hemolytic uremic syndrome
C3G: C3 glomerulopathy
CDC: Complement dependent cytotoxicity
DLBCL: Diffuse large B-cell lymphoma
FB: Complement factor B
FH: Complement factor H
MAC: Membrane attack complex
NHS: Normal human serum
PNH: Paroxysmal nocturnal hemoglobinuria
ΔFB: FB-depleted serum
