# Supplementary material for: Mutations resulting in the formation of hyperactive complement convertases support cytocidal effect of anti-CD20 immunotherapeutics
Source: Cancer Immunol Immunother. 2019 Feb 6;68(4):587–98. doi: 10.1007/s00262-019-02304-0 (PMC6447516; doi:10.1007/s00262-019-02304-0)
Supplement: Supplementary file 1 — Supplementary material 1 (PDF 510 KB) [file 262_2019_2304_MOESM1_ESM.pdf]

Supplementary Fig. 1

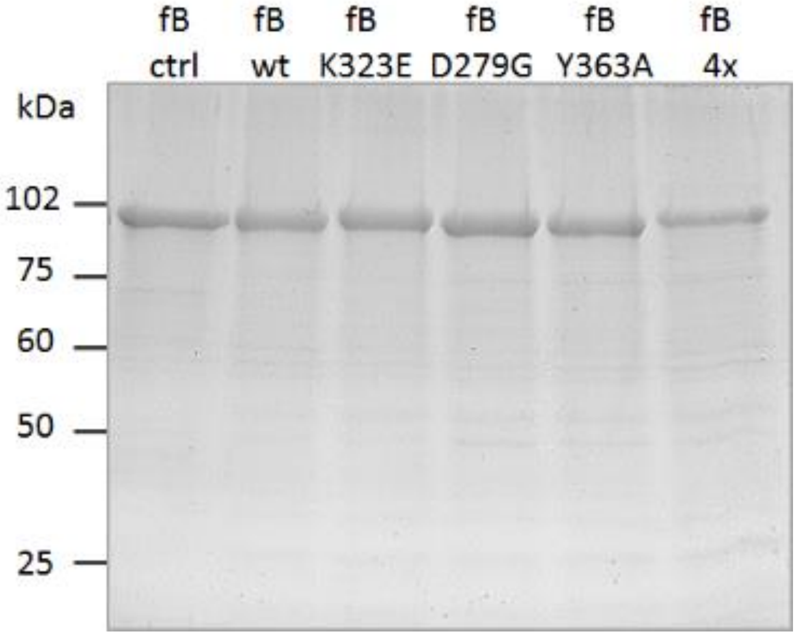

Plasma-purified FB (ctrl), recombinant wild type FB (wt), single (K323E, D279G, Y363A) and quadruple (4x) mutant proteins were electrophoresized on SDS-PAGE gel and stained with Commassie. Lanes were purposely overloaded with 10 μg/ml of protein in order to visualize impurities.

Supplementary Fig. 2

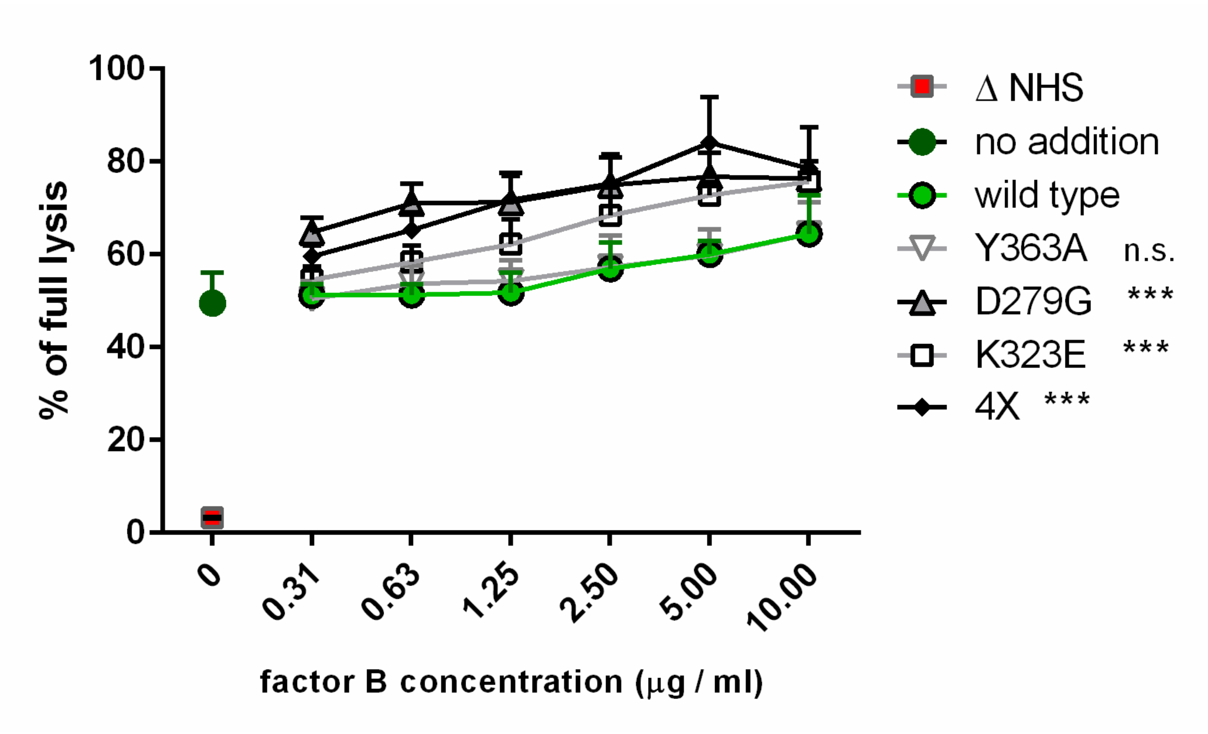

Addition of factor B augments hemolysis of sheep erythrocytes initiated by the classical complement pathway.

Sensitized sheep erythrocytes were mixed with 5% factor B-depleted serum supplemented with recombinant wild type factor B or its single (Y363A, D279G, K323E) or quadruple (4X: Y363A, D279G, K323E, F286L) mutants. Hemolysis was assessed after 30 minutes. The readout obtained for heat-inactivated normal human serum (Δ NHS) represents background lysis. Dunnett's multiple comparison test was applied for classification of differences between lytic curves of particular mutant compared to wild type factor B as: n.s. – not significant or *** - significant at p level < 0.001. Graph shows data from three independent experiments and error bars represent standard deviations.

Supplementary Fig. 3

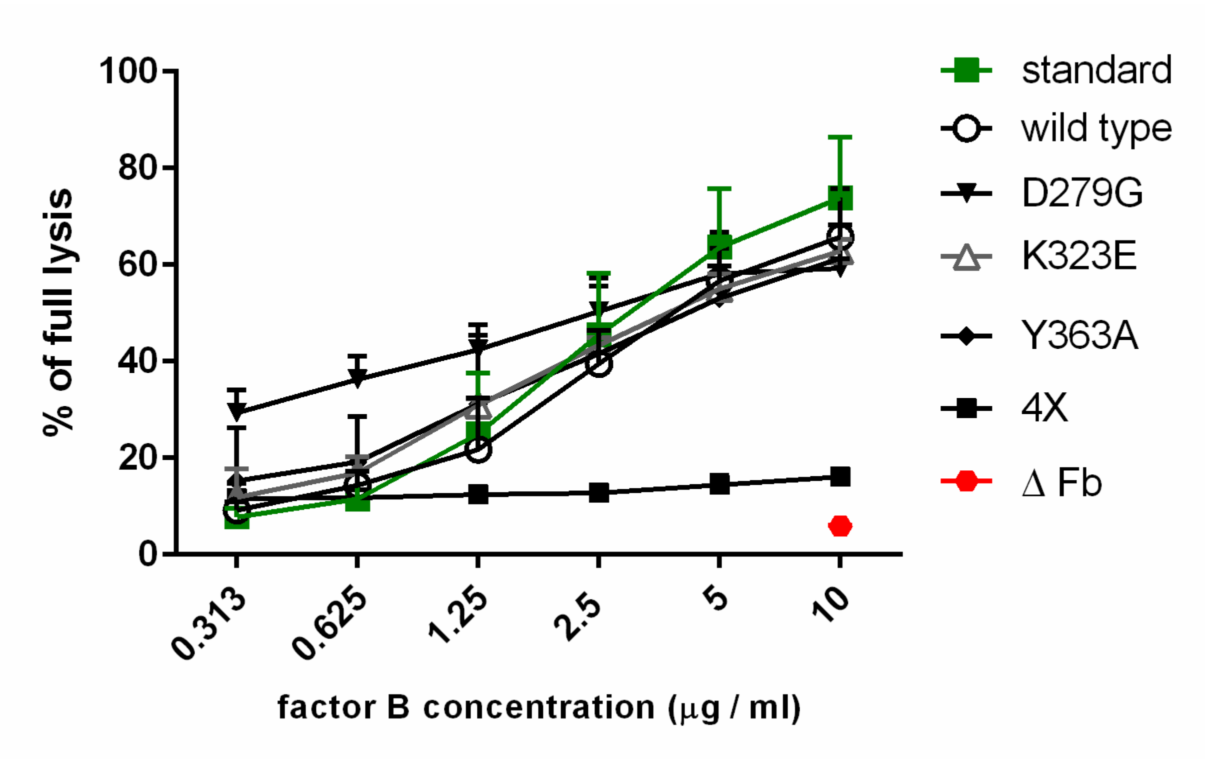

Hemolysis mediated by the alternative complement pathway.

Rabbit erythrocytes were incubated with 5% FB-depleted serum supplemented with increasing concentrations of recombinant wild type or mutated factor B. Plasma purified FB (Complement Technologies) was used as positive control (standard) and FB-depleted serum with no supplementation (Δ FB) served as negative control.

Supplementary Fig. 4

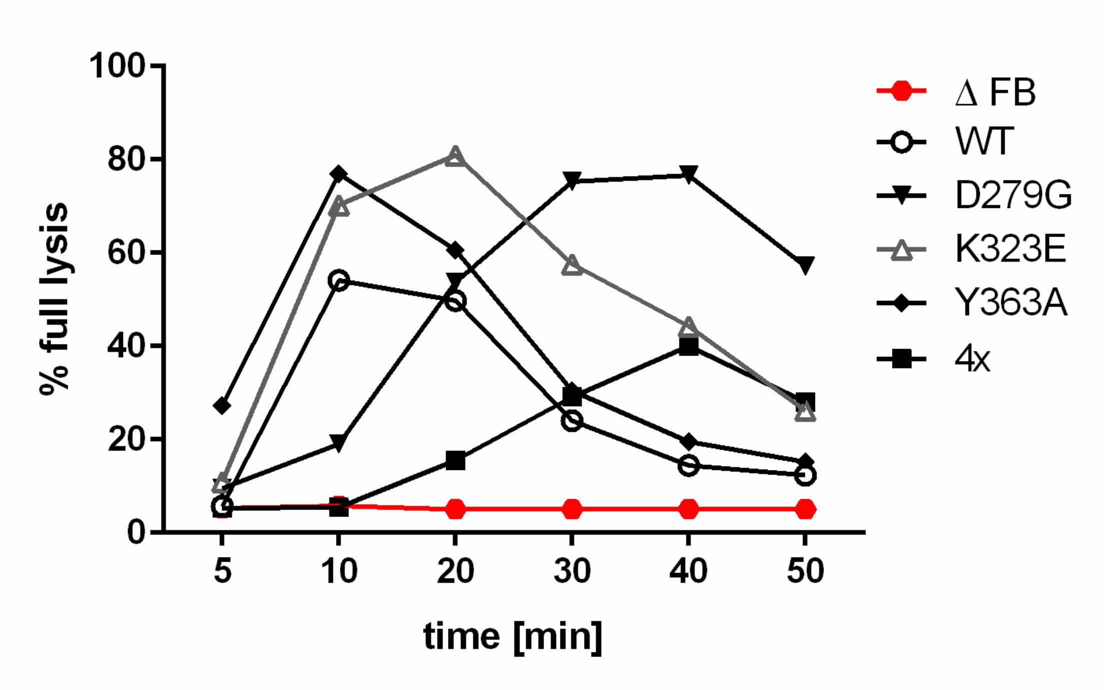

Convertase functional assay performed in FB-depleted serum

Single experiments showing convertase activity in 5% FB-depleted serum (Δ FB) + / - recombinant wild type (WT) or mutated FB variants supplemented at final concentration of 10 μg / ml.
